# Microwave-assisted synthesis of biomass-derived N-doped carbon dots for metal ion sensing

**DOI:** 10.1007/s44246-025-00215-7

**Published:** 2025-06-22

**Authors:** Mehedi Hasan, Balachandran Baheerathan, Shrikanta Sutradhar, Ronak Shahbandinejad, Sudip Rakshit, Janusz Kozinski, Dongbing Li, Yulin Hu, Kang Kang

**Affiliations:** 1 Biorefining Research Institute (BRI) and Department of Chemical Engineering, Lakehead University, Thunder Bay, ON P7B 5E1 Canada; 2 Nottingham Ningbo China Beacons of Excellence Research and Innovation Institute, University of Nottingham, Ningbo, 315104 China; 3 Faculty of Sustainable Design Engineering, University of Prince Edward Island, Charlottetown, PE C1A 4P3 Canada

**Keywords:** Carbon dots, Biomass, Microwave, Heavy metals, Sensing

## Abstract

Biomass-derived carbon dots (CDs) have gained significant research interest for environmental monitoring applications thanks to their cost-effectiveness and sustainability. Using eco-friendly biowastes as precursors for CDs production offers an alternative to expensive and unsustainable inorganic and chemically synthesized CDs. This study presents the findings regarding the successful synthesis of biomass-based nitrogen-doped carbon dots (N-CDs) via a rapid, cost-effective, and environmentally friendly microwave-assisted method. Carboxymethyl cellulose (CMC) and glycine were used as carbon precursors and nitrogen dopants for the first time. The N-CDs exhibited a moderately high quantum yield of 31.6 ± 1.5% with an optimal fluorescence excitation wavelength of 400 nm. FTIR, CHNS, and SEM–EDX analyses characterized the N-CDs' surface functional groups and elemental composition. The optical stability of the N-CDs was validated across varying pH levels and NaCl concentrations. The N-CDs displayed notable selectivity and sensitivity for Fe^3^⁺, Cu^2^⁺, and Hg^2^⁺ ions. The primary quenching mechanisms involve electrostatic interactions, π–π interactions, inner filter effects, and energy transfer. *Stern–Volmer* analysis revealed strong linear quenching for Fe^3^⁺, Cu^2^⁺, and Hg^2^⁺ ions within the 0–10 µM range concentrations, with detection limits (LOD) of 6.0 µM, 1.41 µM and 1.36 µM for Fe^3^⁺, Cu^2^⁺, and Hg^2^⁺, respectively. The fluorescence quenching for Fe^3^⁺ ions enhanced sensitivity at higher concentrations, while selectivity decreased at lower concentrations. These findings highlight the potential of these N-CDs as a cost-effective and sustainable tool for environmental monitoring, offering a promising approach to addressing critical water contamination issues.

**Graphical Abstract:**

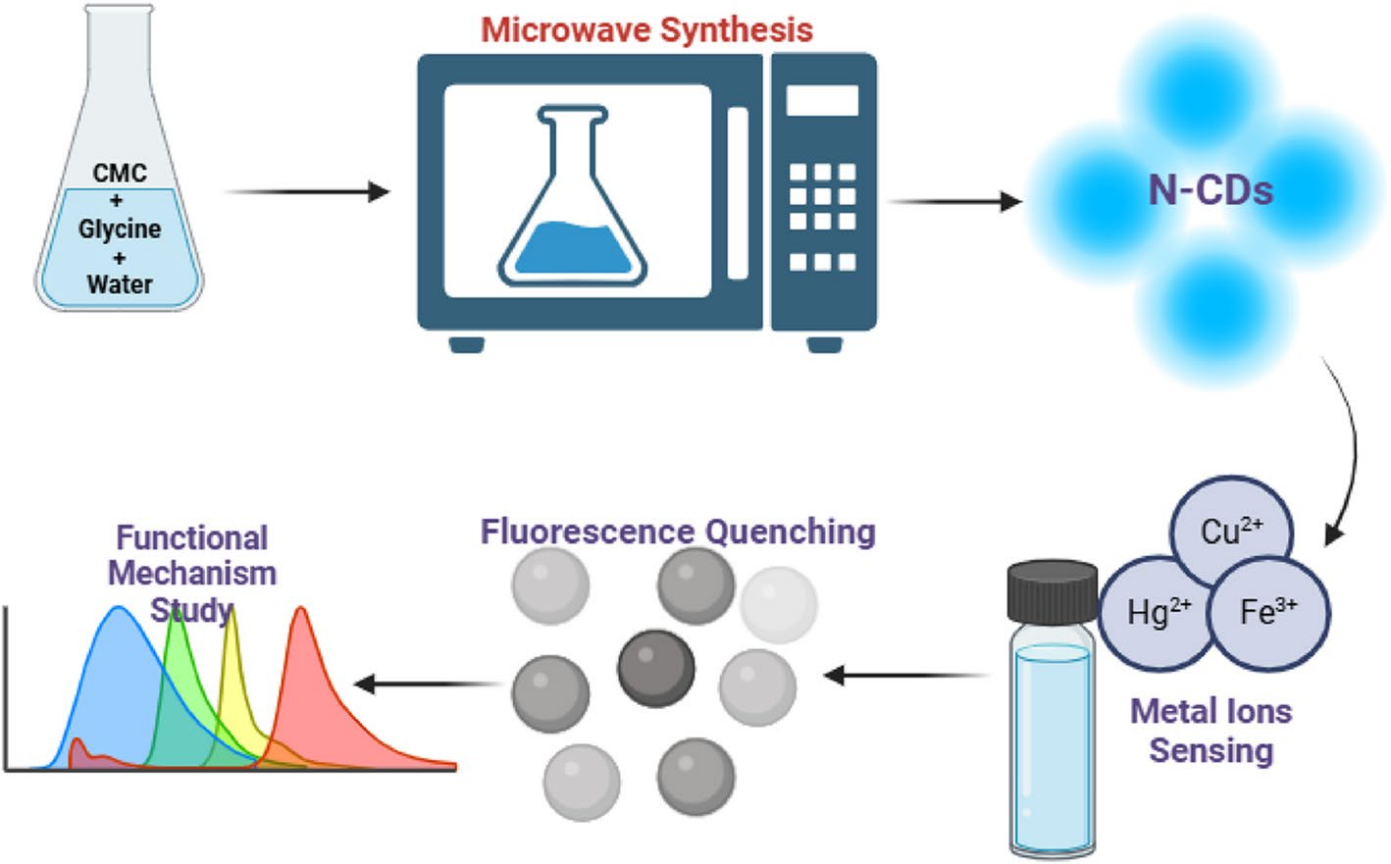

## Highlights

• Biomass-derived N-CDs were synthesized using a rapid microwave-assisted method.

• N-CDs exhibited a high quantum yield of 31.6±1.5% with excitation at 400 nm.

• Selective detection of Fe³⁺, Cu²⁺, and Hg²⁺ ions were achieved via fluorescence quenching.

• Detection limits were 6.0 µM (Fe³⁺), 1.41 µM (Cu²⁺), and 1.36 µM (Hg²⁺).

## Introduction

Heavy metal pollutants in water pose a significant environmental threat to humans. Therefore, rapid and accurate monitoring of the concentration of heavy metal ions in the environment is critical. The traditional spectroscopic methods used for detecting metal ions in polluted water take time and involve complicated sample preparation and analysis procedures. Therefore, developing timesaving, easy-to-apply, sustainable material and metal ion detection procedures is ideal. Carbon dots (CDs) are a relatively new type of luminescent carbon nanomaterial that has gained tremendous research attention for metal ion sensing because of their low cost, easy synthesis, high water solubility, and outstanding biocompatibility. Conventional synthesis methodologies frequently entail using unfavorable commercial chemicals regarding economic viability. Considering these challenges, researchers have been exploring alternative sustainable and eco-friendly routes to fabricate carbon dots, particularly employing biomass-based precursors. Synthesis of Carbon dots based on their core structure can be classified into two main categories: “top-down” method with graphite materials as carbon sources such as laser ablation, electrochemical exfoliation, and chemical oxidation, break down carbonaceous materials into nanoscale carbon dots through physical or chemical processes (Wareing et al. 2021), and “bottom-up” method organic molecules as precursors such as hydrothermal, microwave-assisted, and solvothermal synthesis, which involve the carbonization or decomposition of organic precursors (Li and Li 2021). Among the bottom-up synthesis methods, microwave synthesis is a relatively new approach and was chosen in this study due to its high speed, fast reaction kinetics, selective heating advantage, and the reduced amount of precursor materials required compared to hydrothermal methods. While hydrothermal synthesis is effective, it involves lengthy reaction times and uneven heat distribution, forming larger and thicker particles. In contrast, microwave-assisted synthesis offers a cost-effective and time-efficient alternative, producing CDs with controlled particle size, high yield, and enhanced fluorescence properties, which are essential for sensing applications (Ramezani et al. 2018; Hamed et al. 2024). The uniform heating provided by microwave irradiation reduces particle aggregation and improves surface functionalization, allowing for the incorporation of functional groups such as oxygen, nitrogen, and sulfur. Additionally, CDs synthesized via microwave methods exhibit higher carbon content and larger specific surface areas due to mechanisms such as conductive loss, interfacial polarization, and dipole polarization (Zhao et al. 2025).

Recent investigations have shown the feasibility of synthesizing CDs from a diverse array of biomass-based materials, including carbohydrate-rich biomass resources like cellulose, starch, and chitosan; protein-rich sources such as egg white, silk, and gelatin; lignocellulosic biomass like wood, agricultural residues, and bamboo; as well as various waste materials like lignin, fruit peels, food waste, and industrial by-products (Liang et al. 2013; Gan et al. 2023; Su et al. 2023a, b). These precursors inherently possess carbon, nitrogen, sulfur, and functional groups such as carboxylic, amine groups, etc., therefore, conducive to CD fabrication employing straightforward, cost-effective, and environmentally benign techniques. Additionally, research endeavors have concentrated on fine-tuning the properties of biobased CDs by modulating synthesis parameters like precursor composition, reaction conditions, and surface functionalization strategies (Ishak et al. 2025). Harnessing biobased precursors for carbon dot synthesis epitomizes a sustainable and environmentally conscientious approach. The fluorescence properties of CDs are attributed to factors such as surface states, quantum confinement, conjugated structures, self-trapped excitons, edge defects, free zigzag sites, and multi-emissive centers. Of these, the surface state mechanism has been widely studied. Research indicates that the fluorescence of CDs can be tuned via surface oxidation, functional groups, atom doping, and surface passivation (Liu 2020).

Carboxymethyl cellulose (CMC), a cellulose derivative rich in hydroxyl and carboxyl groups, is an abundant carbon source that can be used for surface-functionalized CDs due to its high reactivity and water solubility (Zhang et al. 2020a, b). Its molecular structure, consisting of β− 1,4-glycosidic bonds and carboxymethyl substitutions (–CH₂COOH), facilitates effective surface functionalization. However, CMC alone lacks nitrogen groups, essential for efficient carbonization, limiting the yield of CDs in microwave-assisted synthesis. On the other hand, glycine, an amino acid rich in amine and carboxyl groups, can act as a nitrogen source to realize the in-situ nitrogen doping and improve the functionalities of the produced CDs. In this regard, the interaction between glycine and CMC’s functional groups is crucial for producing nitrogen-doped CDs with improved optical properties (Cen et al. 2022). Although the synthesis of CDs utilizing CMC has been investigated, CDs with improved characteristics, specifically nitrogen-doped CDs synthesized using glycine, have not yet been reported. Moreover, the application of such CDs in metal ion sensing remains unexplored. Despite the potential of nitrogen doping and functionalized surfaces to enhance sensitivity, selectivity, and detection processes, their effectiveness in this domain is still unexamined. Specifically, selectivity is defined as the ability of a sensor to specifically recognize and respond to a target metal ion, effectively distinguishing it from other interfering ions or molecules in the environment. Sensitivity, on the other hand, is quantified by the lowest detectable concentration of the target metal and is characterized by notable changes in the optical properties of CDs, such as fluorescence intensity or emission wavelength, even at trace metal concentrations. These two parameters are critical for the effective design of CD-based sensors for heavy metal detection (Landa et al. 2022).

The doping and surface functionalization of CDs are vital for heavy metal (HM) sensing, where selective interactions between functional groups and HMs enable effective detection. Doping is an efficient strategy to tailor the optical, electronic, and chemical properties of CDs by incorporating heteroatoms into their carbon framework. Nitrogen doping, widely studied for its ability to tune the energy bandgap, enhances fluorescence quantum yield and photostability. Doping can be achieved through in situ synthesis or post-synthetic treatments, allowing precise control over dopant concentration and distribution to optimize CD functionalities (Wibrianto et al. 2021; Oladzadabbasabadi et al. 2023). Previous studies have demonstrated that heteroatom doping significantly enhances the sensitivity of CDs by increasing their affinity toward specific HMs, making them highly effective for targeted detection and sensing applications (Li et al. 2021; Noun et al. 2021). Biomass-based CDs provide a promising approach for metal sensing applications, due to their unique properties and eco-friendly synthesis processes. CDs are suitable candidates for metal sensing applications as they are highly sensitive and selective probes for identifying and measuring different metal ions such as Hg^2+^, Pb^2+^, Fe^2+^, Cu^2+^, Mn^2+^, Cd^2+^, etc., in complex matrices and aqueous solutions (Huang et al. 2013; Pooja et al. 2019; Qi et al. 2019; Ye at al. 2020; Sariga et al. 2023; Singh et al. 2023; Su et al. 2023a, b). CDs metal sensing capabilities are influenced by particle size, surface chemistry, and environmental conditions (Zhong et al. 2024). Particle size and morphology affect the surface area available for metal interaction, while surface functionalization provides binding sites for metal ions. Optical properties enable signal detection, pH and ionic strength influence metal binding, and interference from matrix components can affect accuracy. Most importantly, surface functional groups on CDs, such as carboxyl, amino, hydroxyl, and thiol groups, can significantly affect their metal sensing capabilities. These functional groups serve as binding sites for metal ions through coordination or electrostatic interactions, thereby influencing the selectivity and sensitivity of metal detection. Despite significant progress being made, CD selectivity for certain metal elements such as Hg^2+^, Cu^2+^, Pb^2^⁺, and Cd^2^⁺ is still not clear. Another gap to mention is that there are not enough assessments on the sustainability of CDs and their toxicity. However, there remains a need to develop more efficient CDs that are cost-effective, easy to produce, and highly practical (Zhao et al. 2020).

To answer the research questions identified above, this study focuses on the microwave-assisted green synthesis of nitrogen-doped carbon dots (N-CDs) utilizing CMC and glycine as precursor materials. This approach emphasizes using biomass-based natural polymers derived from lignocellulosic biomass and amino acids. The physicochemical properties of the synthesized N-CDs, such as their optical characteristics, surface functional groups, and elemental composition, were characterized using various analytical techniques. Furthermore, the resultant N-CDs were employed as an effective"turn-off"fluorescent sensor for detecting heavy metal ions, and their sensitivity and selectivity were thoroughly investigated. Green synthesis, novel biomass-based precursors, and advanced functionality (fluorescent metal sensing) are uniquely combined in this work to create N-CDs with desirable qualities for environmental applications. The originality of this study lies in the simplified and efficient synthesis method, the selection of suitable precursors, and the application of N-CDs in selective heavy metal detection, laying the groundwork for further studies on sustainable nanomaterials. The outcomes of this research will contribute to developing sensor technology that addresses critical water scarcity and contamination challenges.

## Materials and methodology

### Preparation of N-CDs

**Fig. 1:**
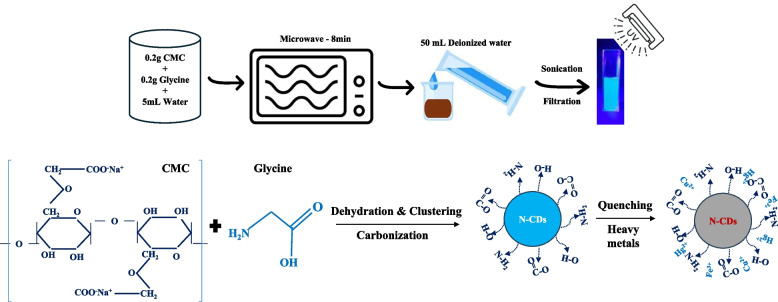
Illustration of microwave-assisted synthesis of N-CDs and heavy metal sensing mechanism

**Fig. 2:**
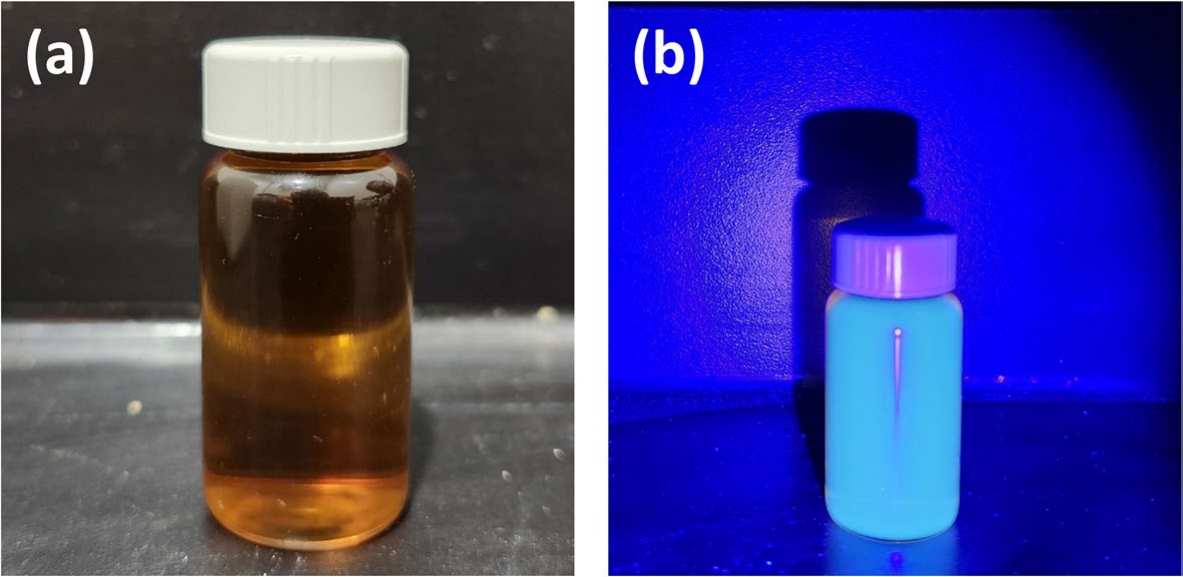
N-CDs solutions exposed to (**a**) ambient light, and (**b**) UV light

All chemicals used are of analytical grade and did not require further purification. Analytical grade CMC and glycine were utilized for N-CDs preparation. Deionized water (DI) was used for all experiments. N-CDs were synthesized by optimizing precursor quantity, solvent volume, and reaction time. Various compositions of CMC and glycine (0.1, 0.2, and 0.3 g each) were tested, along with different solvent volumes (3, 5, 7, and 10 mL of water) and reaction times (5, 8, 10, and 12 min) under full microwave power. The optimal conditions, determined based on fluorescence performance, involved dissolving 0.2 g of CMC and 0.2 g of glycine in 5 mL of deionized water, followed by microwave irradiation for 8 min to induce carbonization. Afterward, 50 mL of deionized water was added, and the mixture was ultrasonicated for 45 min before vacuum filtration. Solid residues were further washed with 30 mL of DI water, and the final CD solution was filtered using a 0.22 µm syringe filter. Initial purification via dialysis membranes resulted in CD loss, so syringe filtration was favored for higher recovery. Figures 1 and 2 are a simplified illustration of the synthesis procedure for N-CDs derived from CMC and glycine and heavy metal sensing mechanisms used in this study.

### Characterization of N-CDs

#### UV–Vis absorbance and fluorescence spectroscopy

UV–Vis absorbance and fluorescence spectroscopies are critical methods for evaluating the optical properties and QY of N-CDs, offering valuable insights into their applications. The UV–Vis absorbance spectra of aqueous N-CD dispersions were recorded using a Cary 5000 UV–Vis-NIR Spectrophotometer (Agilent Technologies) over the 200–700 nm range, with high-resolution data collection at 600 nm/s, 1 nm resolution, and 2 nm bandwidth in a 1 cm quartz cuvette. Fluorescence spectra were captured using a PerkinElmer FL6500 Fluorescence Spectrophotometer, with excitation and emission slit widths of 5 nm and 20 nm, respectively, and an acquisition voltage of 600 V. These techniques were precisely executed to ensure accurate characterization of N-CDs, particularly in terms of their photophysical properties, which are pivotal for various applications. The fluorescence QY is defined as the ratio of photons emitted through fluorescence to photons absorbed, which measures the efficiency of the N-CDs fluorescence (Eq. (1)). To calculate the QY of the synthesized N-CDs, quinine sulfate in 0.1 M H₂SO₄ was used as a reference, excited at 350 nm. The quantum yield of quinine sulfate is well-established at 0.54. The N-CD solutions were diluted 50 times to minimize reabsorption effects, ensuring absorbance below 0.1, which is critical for accurate QY calculations (Schneider et al. 2019).

1 $${QY}_{sample}={QY}_{reference}*\frac{{Integrated\;FI }_{sample}}{{Integrated\;FI }_{reference}}*\frac{{Absorbance }_{reference}}{{Absorbance }_{sample}}*\frac{{\upeta }_{sample}^{2}}{{\upeta }_{reference}^{2}}$$

#### Stern-Vomer relationship for N-CDs and metal ion interactions

The *Stern–Volmer* equation is crucial for understanding the fluorescence quenching behavior of N-CDs in the presence of metal ions, as it quantifies the reduction in fluorescence intensity due to the quencher's interaction. The equation, named after *Otto Stern* and *Max Volmer,* is expressed as

2 $${F}_{0}/F=1+{K}_{sv}\left[Q\right]$$

where *F₀* represents the fluorescence intensity in the absence of the quencher (metal ions), *F* is the fluorescence intensity in the presence of the quencher, *Ksv* is the Stern–Volmer quenching constant, and *[Q]* is the quencher concentration. A linear relationship between *F₀/F* and *[Q]* suggests a simple, dynamic quenching mechanism, typically resulting from collisions between N-CDs and metal ions. However, non-linearity in the *Stern–Volmer* plot implies more complex quenching processes, such as static quenching or a combination of both dynamic and static quenching mechanisms, providing deeper insight into the nature of CD-metal ion interactions. These quenching studies are critical for applications in sensing and environmental monitoring.

#### Material characterization

Fourier transform infrared (FTIR) spectroscopy is a robust technique for characterizing N-CDs, particularly for detecting oxygen-rich functional groups such as hydroxyl, carboxylic acid, amine, imine, and epoxy functionalities. This method provides crucial insights into the surface chemistry of N-CDs, which is important for optimizing their reactivity and performance in various applications. The FTIR analysis was performed using a spectrophotometer (Bruker Tensor 37, Germany) equipped with a Pike MIRacle Diamond Attenuated Total Reflectance (ATR) accessory. Additionally, CHNS analysis of N-CDs offers detailed information about their elemental composition, specifically the levels of carbon (C), hydrogen (H), nitrogen (N), and sulfur (S). This analysis is vital for understanding the N-CDs'doping efficiency, surface functionalization, and overall chemical structure, directly impacting their functional properties and potential applications. The elemental analysis was conducted using an elemental analyzer (Vario EL Cube, Germany). Transmission electron microscope (TEM) imaging experiments were performed on a JEM- 2100Plus transmission electron microscope operated at an acceleration voltage of 200 kV. A Hitachi SU-70 Schottky Field Emission Scanning Electron Microscope (SEM) (Hitachi, Japan), equipped with an Energy Dispersive X-ray Spectroscopy (EDS) detector, was used to examine the morphological characteristics and surface structure of N-CDs. It provides high-resolution images of particle size, shape, and dispersion properties, which are crucial for assessing uniformity and ensuring consistency in their performance across applications. The structural nature of CDs was analyzed using an X'Pert Pro MPD (PANalytical) X-ray diffractometer with Cu Kα radiation (l = 1.5406 Å) at 40 kV. XRD patterns were recorded over a 2θ range of 10–90° at a 2°/min scanning speed. Thermogravimetric analysis (TGA) was conducted to assess the thermal stability of CMC and glycine by monitoring mass changes with temperature. The TGA of individual components and their mixture was performed using a TGA-i1000 Instrument Specialist under a nitrogen (N₂) atmosphere. The analysis was conducted from 30 °C to 700 °C at a heating rate of 10 °C min⁻^1^.

## Results and discussion

### Structural and morphological properties of N-CDs

**Fig. 3:**
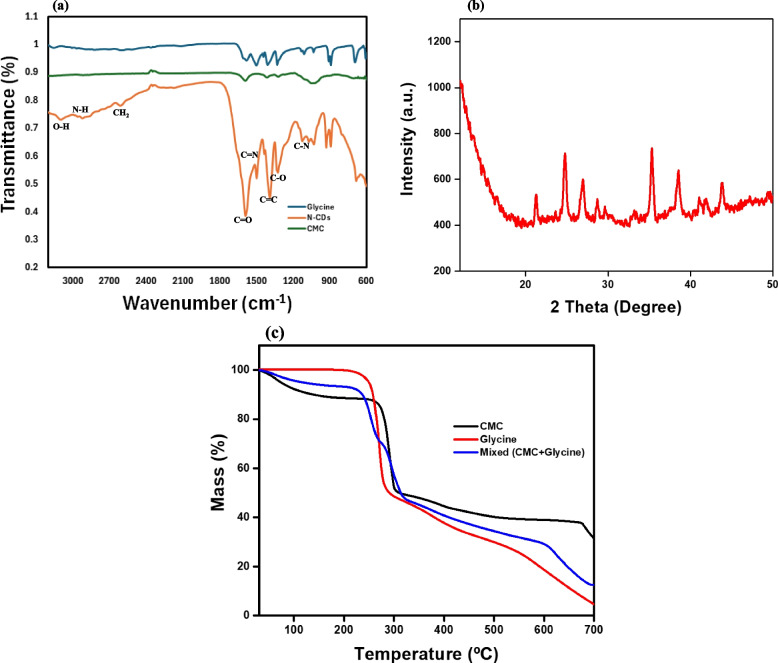
**a** FTIR spectrum of N-CDs prepared from biomass (CMC and Glycine) by microwave irradiation, (**b**) XRD patterns for N-CDs, and (**c**) Thermograms of the CMC, glycine, and mixed precursors

Figure 3a presents the FTIR spectra of the N-CDs, CMC, and Glycine, showing their functional groups. The spectrum of the N-CDs exhibits characteristic broad absorption bands at 3130 cm⁻^1^ and 3028 cm⁻^1^, corresponding to O–H and N–H stretching vibrations, respectively. Additionally, peaks observed around 1600 cm⁻^1^ and 1100 cm⁻^1^ were attributed to C=N and C–N bond vibrations, respectively, further verifying the incorporation of these functional groups in the N-CDs. These findings provide solid evidence of effective synthesis and functionalization of the N-CDs, which will be crucial for enhancing the CDs performance in metal sensing applications. Architha et al. (2021) reported similar findings in their study on the microwave-assisted green synthesis of fluorescent carbon quantum dots (CQDs) from Mexican mint extract for Fe^3^⁺ detection and bio-imaging. They identified a peak at 3276 cm⁻^1^ associated with the O–H group on the CD surface, along with C–H stretching at 2874 cm⁻^1^, C–O stretching at 1581 cm⁻^1^, and C–C stretching at 1406 cm⁻^1^. A peak at 1066 cm⁻^1^ corresponded to the bending vibration of the C–O group, while peaks at 934 cm⁻^1^ and 661 cm⁻^1^ were linked to aromatic sp^2^ bending. Similarly, Lu et al. (2018) reported comparable peaks for CQDs from watermelon juice, with characteristic peaks at 2854 cm⁻^1^ and 1056 cm⁻^1^ related to C–H stretching and C–O groups. Additionally, Chauhan et al. (2020) observed C–H stretching and CH₂ bending at 2960 cm⁻^1^ and 1450 cm⁻^1^, respectively, in CQDs synthesized from coconut coir, along with an aromatic sp^2^ bending peak at 650 cm⁻^1^.

In Fig. 3b, the XRD analysis reveals a complex diffraction pattern with distinct peaks at approximately 21.26°, 24.76°, 27.01°, 35.33°, 38.52°, and 43.92°. Bragg’s equation was employed to calculate the corresponding d-spacing values for the CDs, which were found to be 4.18 Å, 3.59 Å, 3.30 Å, 2.54 Å, 2.34 Å, and 2.06 Å, respectively. These diffraction peaks correspond to the (0 0 2), (1 1 0), (1 0 0), and (1 0 1) planes of graphitic carbon (Yalshetti et al. 2024). The sharpness and intensity of these peaks indicate that N-CDs possess a polycrystalline structure (Su et al. 2023a, b; Yalshetti et al. 2024). Unlike graphene quantum dots, which typically exhibit a peak around 27°, the multiple peaks in the N-CD pattern suggest minor structural variations. In previous studies, Similar XRD patterns have been reported in the microwave-assisted synthesis of CDs, further supporting the presence of structural heterogeneities in the synthesized N-CDs (Mintz et al. 2021; Yalshetti et al. 2024). Figure 3c presents the TGA curves for CMC, glycine, and their mixture over a temperature range of 30 °C to 700 °C. In all samples, the initial weight loss observed between 30 °C and 230 °C is attributed to the evaporation of free water and dehydration reactions. Previous research (Tischer et al. 2019) also suggests that this mass loss may be partly due to the formation of ammonia gas resulting from –NH₂ groups. Pure CMC and glycine exhibit single-stage decompositions at approximately 260 °C and 230 °C, respectively, reflecting the degradation of their primary structures. In contrast, the CMC and glycine mixture shows a two-stage weight loss; the first stage, between 230 °C and 270 °C, corresponds to the degradation of glycine, while the second stage, between 265 °C and 315 °C, is associated with the decomposition of the CMC main chains. Mintz and coauthors (2021) stated that the mass loss during these stages is attributable to the decomposition of C=O and/or –COOH groups. Lastly, the stage from 330 °C to 658 °C corresponds to the decomposition of sp^3^ carbon or other organic-like structures in N-CDs cores.

**Fig. 4:**
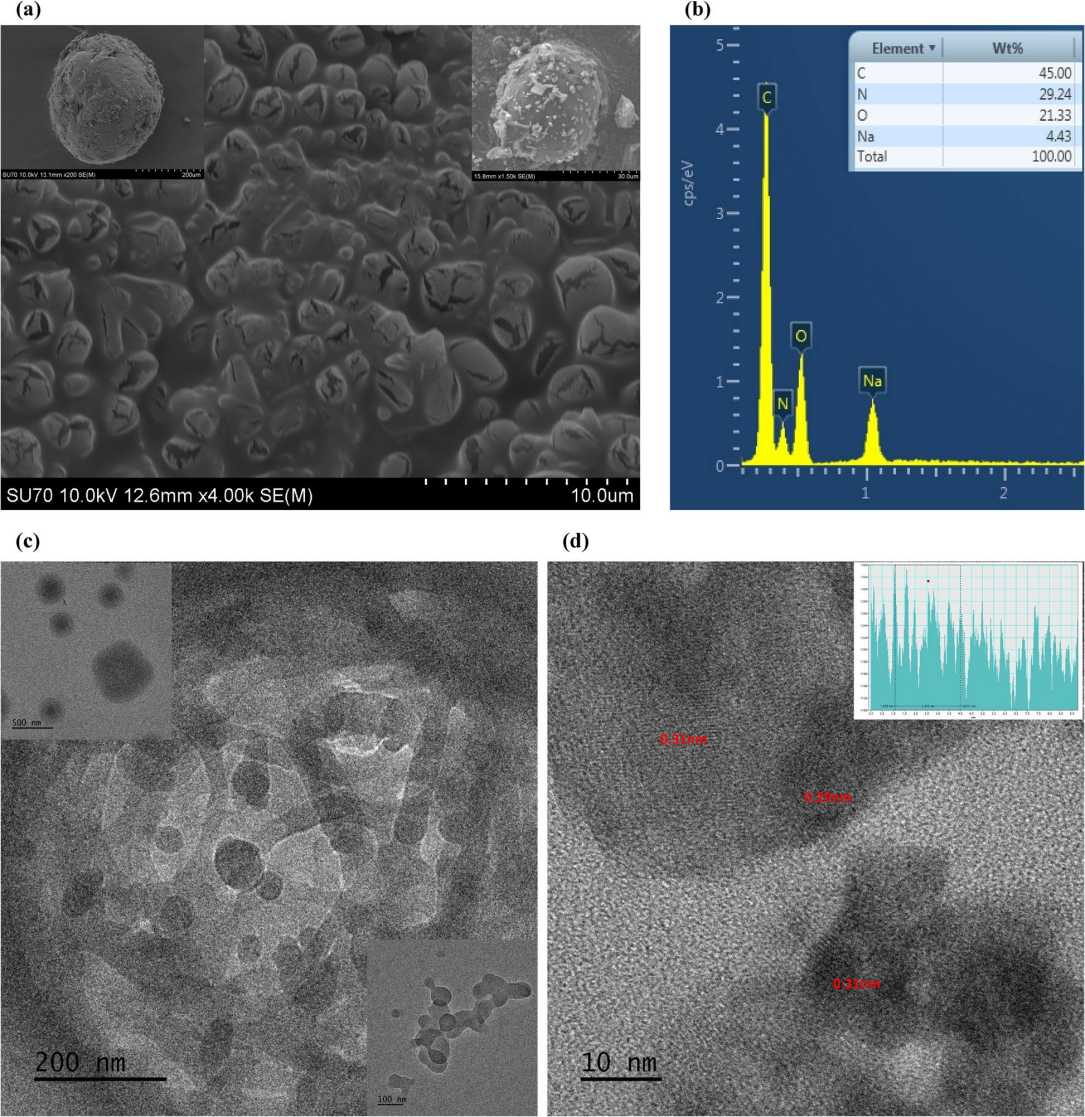
**a** SEM images of N-CDs, (**b**) EDX spectrum of N-CDs, (**c**) TEM images at various magnifications, and (**d**) lattice fringes

The CHNS analysis results, presented in Table 1, revealed that the CDs contain 13.08% N, confirming the successful incorporation of nitrogen functional groups during synthesis. During the metal-detecting applications, the functionality and performance of the CDs are impacted by their elemental compositions. Through lone pair electrons, the nitrogen groups increase the availability of metal-binding sites, enabling robust coordination with metal ions and enhancing selectivity and sensitivity. Additionally, nitrogen groups alter the CDs electrical structure, resulting in optical characteristics that may be adjusted, such as the improvement or quenching of fluorescence, which are essential for detecting systems. It is also recognized that the strong electronegativity of nitrogen functional groups in carbon networks attracts and captures lithium polysulfides and provides active sites for alkaline ions, especially lithium ions (Lee et al. 2024). Furthermore, nitrogen doping improves the CDs hydrophilicity and dispersibility, facilitating improved metal-ion interaction in aqueous solutions. The elemental composition analysis of the N-CDs revealed (Table 1) the carbon content of 35.78%, which highlights the primary carbonaceous framework originating from the CMC, a carbon-rich precursor. The nitrogen content of N-CDs was measured at 13.08%, confirming effective nitrogen doping attributed to glycine as the nitrogen source. Additionally, the hydrogen content of 4.97% indicates the incorporation of hydrogen atoms bonded to carbon and nitrogen, likely within diverse functional groups contributing to the N-CDs structural and functional properties. The SEM image confirms that the N-CD is spherical and has a rough texture (Fig. 4a). Similar physical properties were reported by Jlassi et al. (2020), where CDs were synthesized from petroleum coke waste via thermal treatment in the presence of ammonia. The EDX analysis results (Fig. 4b) indicate that the nitrogen-doped carbon dots (N-CDs) consist of approximately 45% carbon (C), 23% nitrogen (N), and 23% oxygen (O), confirming the successful incorporation of nitrogen into the carbon dot structure. The absence of detectable impurities further supports the purity and controlled synthesis of the N-CDs. However, slight variations in elemental composition compared to CHNS analysis may arise due to methodological differences, as EDX provides localized elemental data from specific points, whereas CHNS analysis offers bulk compositional information. The TEM results, presented in Fig. 4c, provide insights into the surface morphology of the synthesized N-CDs at different magnifications and reveal nearly spherical; however, significant particle overlap is observed, making individual separation challenging, like SEM observations. The samples were dried using the freeze-drying method, which may have led to particle aggregation, potentially affecting the observed morphology. Moreover, high-magnification images in Fig. 4d reveal distinct lattice orientations with an inter-lattice spacing of 0.29 nm and 0.31 nm, indicating a dominant crystalline structure. Conventional studies often report an inter-lattice spacing of 0.21 nm, corresponding to the (100) plane of graphite. However, the slight disparity observed in this study can be attributed to incorporating nitrogen atoms within the lattice structure, leading to electrostatic repulsion, consistent with previous reports on doped CDs (Ibarra-Prieto et al. 2023).

**Table 1:** Elemental composition of N-CDs

Sample	N%	C%	H%	O%
N-CDs	13.08	35.78	4.97	46.17
CMC	0.00	35.20	6.44	58.36
Glycine	18.95	32.03	8.41	40.61

### Optical properties of N-CDs

#### N-CDs FL intensity at a different excitation wavelength

**Fig. 5:**
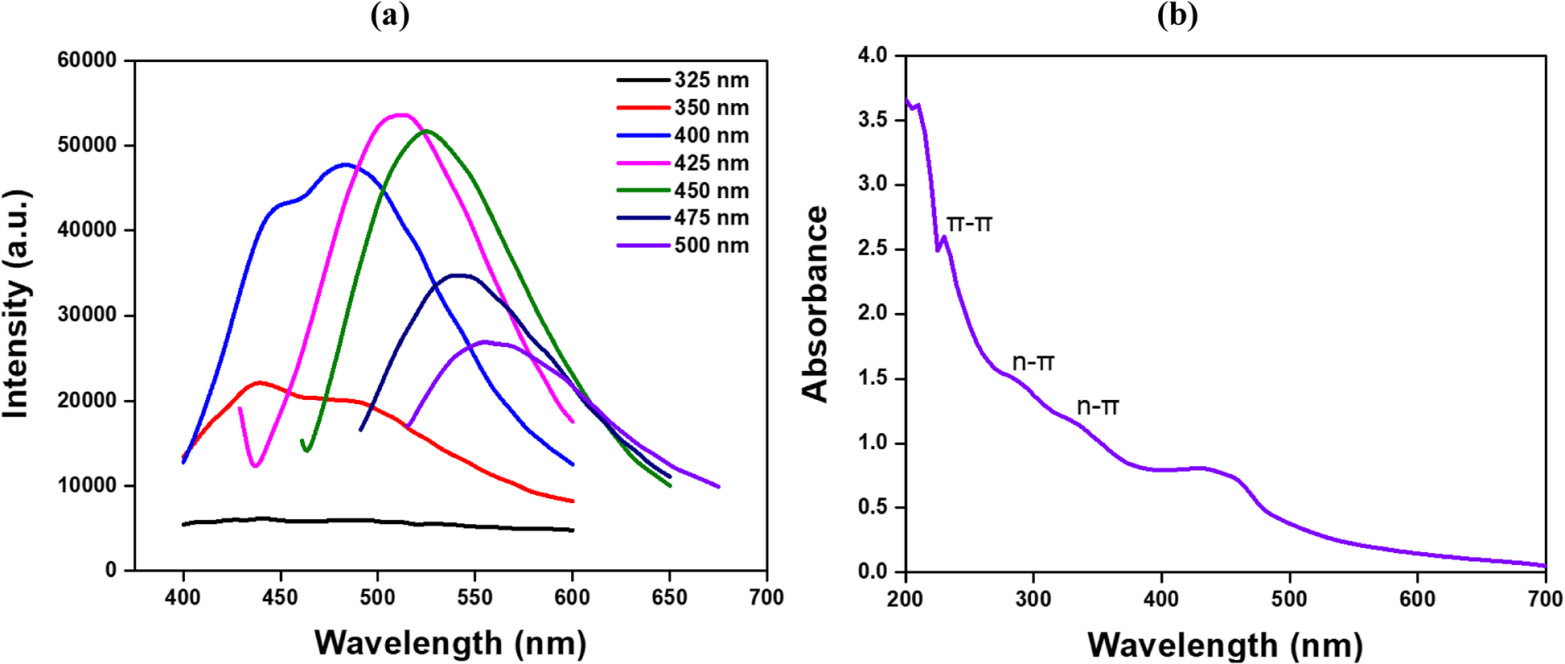
N-CDs (**a**) FL intensity at different excitation wavelengths, and (**b**) UV–vis absorption spectra

Figure 5b presents the UV–vis spectra of the synthesized N-CDs, revealing two distinct absorption regions. The peak in the 200–300 nm range corresponds to the π–π* transition of C = C bonds, indicating aromatic sp^2^ domains within the carbon core (Zhan et al. 2019; Architha et al. 2021). Meanwhile, the peak in the 300–400 nm range is usually ascribed to the n–π* transition, which originates from the carboxyl functional groups on the surface of the fluorescent carbon dots (Yue et al. 2019). A low-energy tailing is observed in the N-CDs beyond 500 nm. Architha et al.’s (2021) study on microwave-assisted green synthesis of fluorescent CQDs from Mexican mint extract has reported similar absorption features. This data reveals important insights into the molecular structure and bonding properties of the N-CDs, particularly the heteroatom-containing π-electronic bands within the aromatic sp^2^ domains and the overall structural order of the system (Mintz et al. 2021; Atchudan et al. 2024).

When photons excite CDs, their electrons move to higher energy states and then return to the ground state, releasing excess energy as light through a process known as photoluminescence (PL). This property is a defining feature of CDs and plays a vital role in their optical behavior. In fluorescence-based sensors, changes in fluorescence (FL) enable the detection of target analytes, such as heavy metals. Therefore, understanding the FL properties of the N-CDs is the key to developing effective and reliable sensing strategies (Li and Li 2021). N-CDs FL intensity was analyzed at various excitation wavelengths from 325 to 500 nm to determine the optimal conditions for metal ion sensing, as illustrated in Fig. 5a. The results indicated that no significant emission peak was observed at excitation wavelengths between 325 and 350 nm. However, a sharp emission peak centered at 500 nm was detected when excited between 375 and 450 nm, with maximum intensity achieved at 500 nm due to n − π* transitions (Ayad et al. 2023). An excitation wavelength of 400 nm was chosen for further studies due to its higher intensity, making it suitable for examining the quenching effect in the presence of metal ions. This excitation-dependent emission behavior was attributed to factors such as the surface states of C–O bonds and polydispersity, as confirmed by FTIR analysis. Experiments were conducted at neutral pH (~ 7) to investigate the interaction between CDs and metal ions. Two concentrations of metal ions were selected to evaluate their quenching effect on the fluorescence intensity of CDs, providing insights into the sensitivity and response of CDs for metal ion detection. Similar findings were reported by Architha et al. (2021), who investigated the photoluminescence properties of Carbon Quantum Dots (CQDs) derived from biomass, exciting the samples at wavelengths ranging from 320 to 440 nm. They observed a sharp emission peak at 436 nm with excitation wavelengths between 320 and 380 nm, reaching maximum intensity at 340 nm. Additionally, solvent polarity was noted to influence the emission wavelength shift. Other studies have reported comparable blue-emitting carbon dots; for instance, Bajpai et al. (2019) noted similar results from milk protein, while Huang et al. (2018) found a maximum emission wavelength of 442 nm for CQDs derived from Bauhinia flowers at 355 nm excitation. Jia et al. (2019) observed a peak at 470 nm with 360 nm excitation from black soya beans and Shen et al. (2017) reported a peak at 442 nm from sweet potato, with redshifts from 406 to 486 nm at varying excitation wavelengths.

#### Quantum yield (QY) of N-CDs

The quantum yields (QYs) of CDs can be increased through heteroatom doping, which significantly improves the sensitivity of CD-based sensors for heavy metal detection because higher quantum yields indicate that a larger proportion of absorbed photons are converted into observable optical signals, such as fluorescence. In this study, the QY of the N-CDs was determined using quinine sulfate in 0.1 M H₂SO₄ as a reference, with a known QY of 0.54 (Melhuish 1960). Equation (1) was applied for the QY calculation. The N-CD solution was diluted 50 times to minimize reabsorption effects, keeping the absorbance below 0.1 (Schneider et al. 2019). N-CDs absorbance was measured at 0.09, with an integrated fluorescence intensity (I) of 118,041,300. Then, the quantum yield was determined by replacing the corresponding values in Eq. (1), yielding a QY of 31.6 ± 1.5%. This substantial QY demonstrates the effective photon emission capability of N-CDs, highlighting their potential for applications in fluorescent sensing and imaging. Architha et al. (2021) reported a QY of 17% for CQDs synthesized from Mexican mint leaves. Table 2 presents a comparative analysis of CDs synthesized from various biomass sources, emphasizing their properties and effectiveness in detecting metal ions. The CDs produced in this study demonstrated a moderately higher quantum yield than those synthesized using other precursors and methods. This enhancement can be attributed to particle size, surface functional groups, and heteroatom doping, which influence the fluorescence emission mechanism. Although the LOD requires further improvement, it meets the threshold established by WHO standards.

**Table 2:** Comparison of the properties of CDs prepared from various biomass and their ability to detect Cu^2+^ , Fe^3+^and Hg^2+^ions

*Source of CDs*	*Synthesis Method*	*Quantum Yield (%)*	*Limit of Detection (LOD)*	*Metal Ion Detected*	*Reference*
*Lily bulbs*	Microwave	17.60%	0.013 µM	Cu^2+^	(Gu et al. 2018)
*Ammonium citrate and triethylenetetramine*	Microwave	29.83%	0.0045 µM	Cu^2+^	(Liu et al. 2019a, b)
*Ascorbic acid and urea*	Hydrothermal	7.00%	0.15 µM	Cu^2+^	(Bhatt et al. 2020)
***CMC and Glycine***	***Microwave***	***31.60%***	***1.41 µM***	***Cu***^***2*+**^	***This study***
*Brewer's spent grain*	Microwave	-	0.095 µM	Fe^3+^	(Deng et al. 2021)
*Roasted chickpeas*	Microwave	1.80%	2.74 μM	Fe^3+^	(Başoğlu et al. 2020)
*Coccinia grandis*	Hydrothermal	17.50%	0.53 μM	Fe^3+^	(Atchudan et al. 2024)
*Elm seeds*	Hydrothermal	6.15%	3.18 μM	Fe^3+^	(Zhang et al. 2022)
***CMC and Glycine***	***Microwave***	***31.60%***	***6.0 µM***	***Fe***^***3*+**^	***This study***
*Ascorbic acid and urea*	Microwave	7.00%	0.20 µM	Hg^2+^	(Bhatt et al. 2020)
*Gum ghatti*	Microwave	23.00%	0.004 µM	Hg^2+^	(Sekar et al. 2022)
***CMC and Glycine***	***Microwave***	***31.60%***	***1.36 µM***	***Hg***^***2*+**^	***This study***

### Evaluating N-CDs fluorescence detection sensitivities of different metal ions

**Fig. 6:**
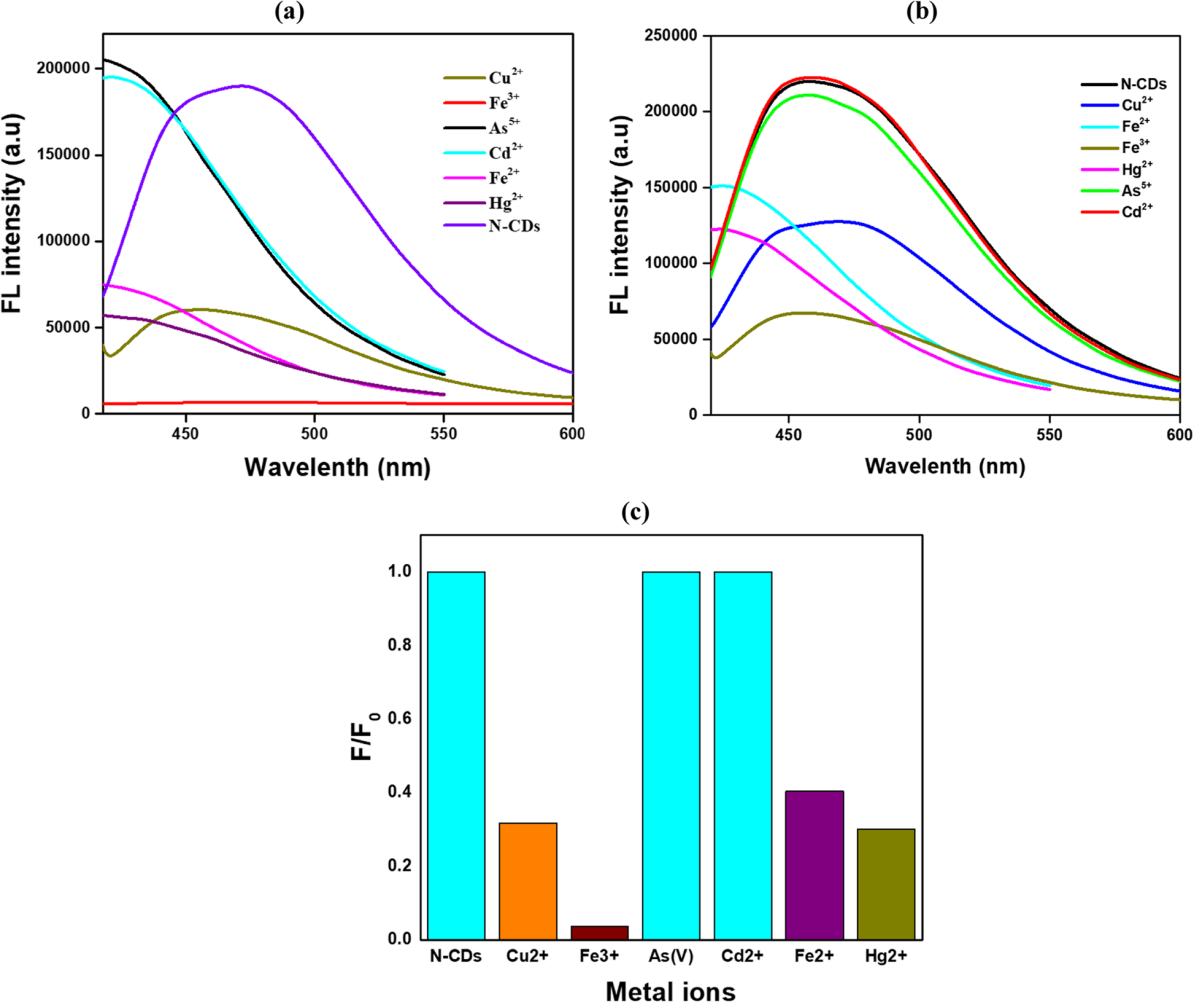
**a** N-CDs fluorescence quenching with 0.05 M metal ions, (**b**) 0.001 M metal ions, and (**c**) relative FL response difference between blank N-CDs and N-CDs in metal ion solutions

Although numerous analytical techniques are available for detecting and quantifying metal ions in water, using CDs as sensing probes offers a facile and cost-effective alternative. Various metal ions were tested under the same conditions to examine the specificity of N-CDs. Figure 6a, b illustrates the fluorescence (FL) spectra of N-CDs exposed to various metal ions, including As^5^⁺, Fe^3^⁺, Fe^2^⁺, Hg^2^⁺, Cu^2^⁺, Zn^2^⁺, and Cd^2^⁺, at different concentrations to investigate their fluorescence quenching behavior. The N-CD solution exhibited a maximum emission intensity at 470 nm under blank conditions, which progressively diminished upon adding these metal ions. Among the tested ions, the N-CDs demonstrated the highest selectivity and quenching efficiency for both concentrations for sensing Fe^3^⁺. This phenomenon was attributed to the ion-specific interaction between Fe^3^⁺ and the functional groups on the N-CD surface and their charge selectivity. Figure 6c presents the fluorescence intensity ratio (F₀/F) of N-CDs before and after adding various metal ions, with F₀ and F representing fluorescence intensities in the absence and presence of metal ions, respectively. Significant fluorescence quenching was explicitly observed for Fe^3^⁺, Fe^2+^, Hg^2^⁺, and Cu^2^⁺ ions, whereas other metal ions exhibited no alterations in the fluorescence signal. Similar findings were reported by Architha et al. (2021), who demonstrated a quenching hierarchy for metal ions, where Fe^3^⁺ exhibited the most substantial quenching effect, followed by Mn^2^⁺ > Cu^2^⁺ > Zn^2^⁺ > Pb^2^⁺ > Ni^2^⁺, with Ni^2^⁺ ions producing the least quenching. Likewise, Zhang et al. (2020a, b) reported comparable experiment results.

### Detailed study of fluorescence quenching of Fe^3+^ ions

Fe is a vital element for life, usually existing in two oxidation states, ferrous (Fe^2^⁺) and ferric (Fe^3^⁺). Among these, Fe^3^⁺ at pollutant concentration is particularly harmful due to its low solubility and ability to produce toxic radicals. Excess Fe^3^⁺ in the environment or the human body is linked to severe health issues, including Parkinson’s disease, cytotoxicity, and metabolic disorders. Its strong electron-accepting tendency can disrupt cellular systems (Singh et al. 2020). According to World Health Organization (WHO) guidelines, the maximum permissible Fe^3^⁺ concentration in drinking water is 5.36 mM (Organization 2022). Consequently, there is growing interest in developing simple, portable, and cost-effective fluorescent CD-based devices for the selective and sensitive detection of Fe^3^⁺ (Landa et al. 2022). Since our N-CDs showed good potential in Fe detection, a further detailed study regarding fluorescence quenching of Fe^3+^ was conducted in this study.

**Fig. 7:**
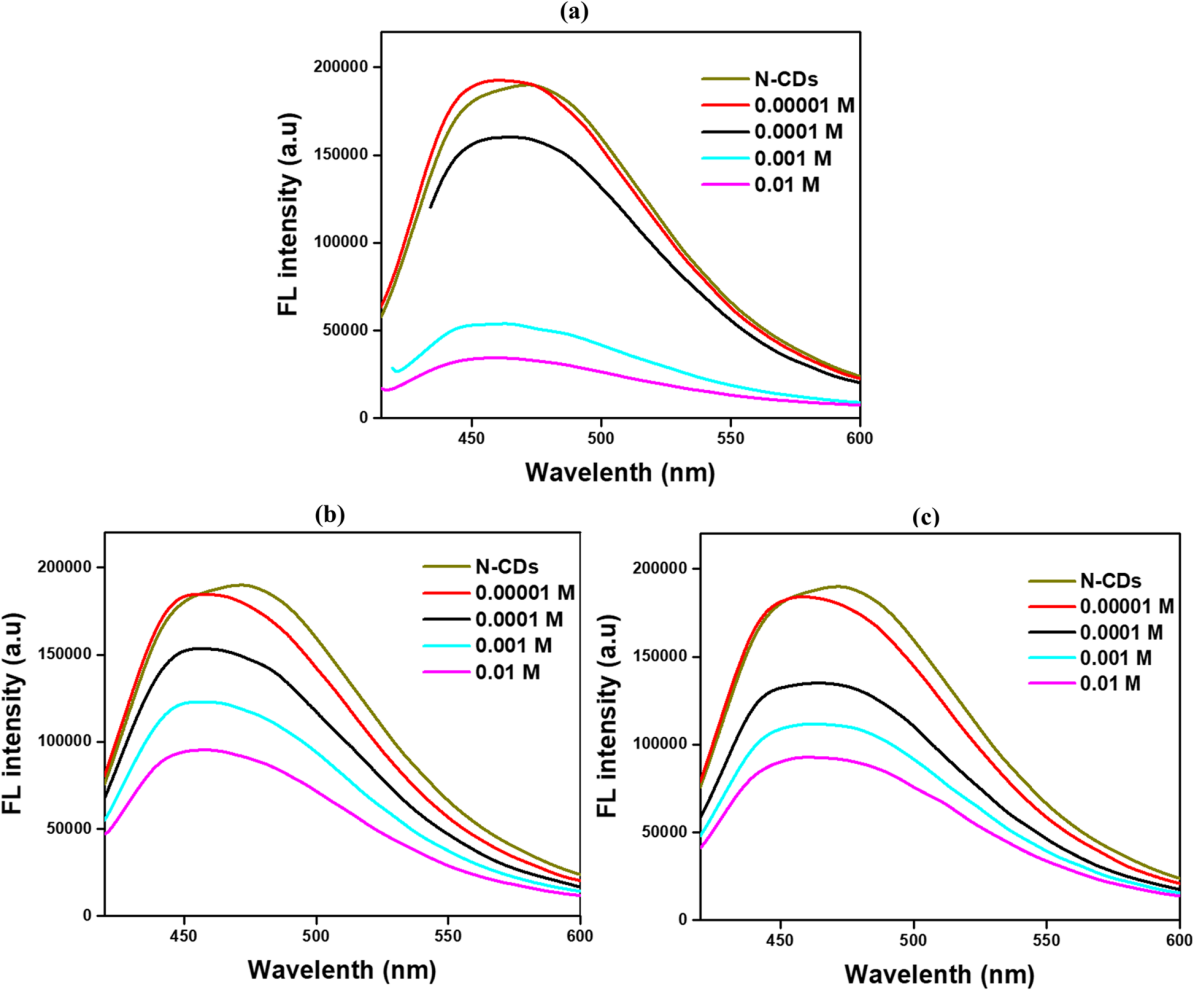
N-CDs fluorescence quenching of (**a**) Fe^3+^, (**b**) Cu^2+^, and (**c**) Hg^2+^ ions at different concentrations

The fluorescence quenching of N-CDs using Fe^3^⁺ at varying concentrations was examined, as shown in Fig. 7a. Fe^3^⁺ ions exhibit absorption bands that overlap with the excitation wavelength of the N-CDs, leading to a notable reduction in the observed fluorescence intensity, a phenomenon known as the Inner Filter Effect (IFE). At higher concentrations of Fe^3^⁺ ions, this effect significantly reduced the fluorescence intensity of the N-CDs; however, at lower concentrations, the quenching effect becomes less pronounced, resulting in a loss of selectivity (Zhang et al. 2020a, b). Literature suggests that the fluorescence quenching of CDs by Fe^3^⁺ is primarily due to electron transfer and the interaction of oxygen-containing functional groups on the CD surface, which modify surface energy states and promote non-radiative recombination pathways (Das et al. 2019; Atchudan et al. 2020; Architha et al. 2021). Fe^3+^ ions show high absorption bands that overlap with the CDs excitation wavelength, which causes the IFE. Specifically, the CDs receive less excitation light because of this overlap, lowering their fluorescence intensity. Significant fluorescence quenching and a noticeable reduction in the brightness of the produced light are the results of the effect, which is more visible at larger Fe^3^⁺ concentrations. Fluorescence is further reduced by this interaction, which stops the CDs from emitting photons and returning them to their ground state. These mechanisms impact both the color shift and the efficiency of sensing. Because quenching reduces the strength of the released light, it may cause a shift in the color of visible fluorescence. Due to mild quenching, selectivity is diminished at lower concentrations of Fe^3^⁺, making it more difficult to distinguish Fe^3^⁺ from other ions. However, the evident quenching at higher concentrations (0.01M) means the N-CDs are still capable of detecting metal ion pollutants when their concentrations are high (Architha et al. 2021). A previous study of N-CDs synthesized from rice residue and glycine demonstrated selective fluorescence quenching for Fe^3^⁺ detection in actual water samples due to coordination between phenolic hydroxyl groups on the N-CDs surface and Fe^3^⁺ ions (Qi et al. 2019). Similarly, N-CDs produced from the banana peel with aqueous ammonia exhibited fluorescence quenching via interactions between Fe^3^⁺ and surface functionalities (-COOH, -OH, -NH₂) (Atchudan et al. 2020). Kalanidhi and Nagaraj (2021) reported that N-CDs synthesized using betel leaves exhibited fluorescence quenching by Fe^3^⁺ through static and dynamic mechanisms, highlighting their applicability in environmental and biological contexts. To increase selectivity and sensitivity and guarantee their effectiveness in biological and environmental applications, this dual behavior emphasizes the significance of optimizing the concentration range for Fe^3+^ detection and producing CDs with certain optical properties (Lu et al. 2018).

### Fluorescence quenching of Hg^2+^ and Cu^2+^ ions at different concentrations

Mercury (Hg^2^⁺) and copper (Cu^2^⁺) pollution are also critical environmental concerns due to their toxicity and potential for bioaccumulation. Hg^2^⁺, a non-biodegradable metal ion, poses severe risks to living organisms and ecosystems, with even minimal accumulation in aquatic food chains. While Cu^2^⁺ is essential for biological processes, as it forms part of the respiratory enzyme cytochrome c oxidase, excessive exposure can cause acute gastrointestinal issues and chronic damage to the liver and kidneys (Zulfajri et al. 2019; Landa et al. 2022). N-CDs fluorescence quenching behavior was systematically evaluated across spectra of Cu^2^⁺ and Hg^2^⁺ concentrations, as illustrated in Fig. 7b, c. The emission intensity of N-CDs gradually quenched with the increase of Cu^2^⁺ and Hg^2^⁺ concentration. At lower ion concentrations, both Cu^2^⁺ and Hg^2^⁺ induced comparable reductions in the fluorescence intensity of the CDs. These results indicate that the quenching mechanisms for Cu^2^⁺ and Hg^2^⁺ are similar under identical experimental conditions. Previous studies have highlighted using N-CDs for Hg^2^⁺ detection in water samples. Gu et al. (2016) synthesized N-CDs from lotus root using microwave-assisted synthesis, demonstrating their ability for multicolor fluorescence bioimaging and environmental testing, with Hg^2^⁺ sensing driven by the formation of CDs-Hg^2^⁺ complexes. Similarly, Liu et al. (2019a, b) developed multi-emission fluorescent CDs from bamboo leaves for detecting Hg^2^⁺ in complex river water. Xie et al. (2019) reported that N-CDs derived from Highland Barley exhibited fluorescence quenching due to the chelation of Hg^2^⁺ with carboxylic groups on the N-CDs surface. Pineapple peel-based CDs also effectively detected Hg^2^⁺ in tap and lake water, with a decrease in fluorescence lifetime, indicating electron transfer upon Hg^2^⁺ interaction (Vandarkuzhali et al. 2017). Additionally, Das et al. (2017) prepared N-CDs from lemon juice and l-arginine to detect Cu^2^⁺ in river water. The sensing mechanism was further explored by adding EDTA to the quenched Cu^2^⁺-CDs complex, which fully restored the fluorescence signal, eliminating the inner filter effect of the cupric amine complex.

### Determination of limits of detection (LOD) of Fe^3+^, Cu^2+^ and Hg^2+^ ions

**Fig. 8:**
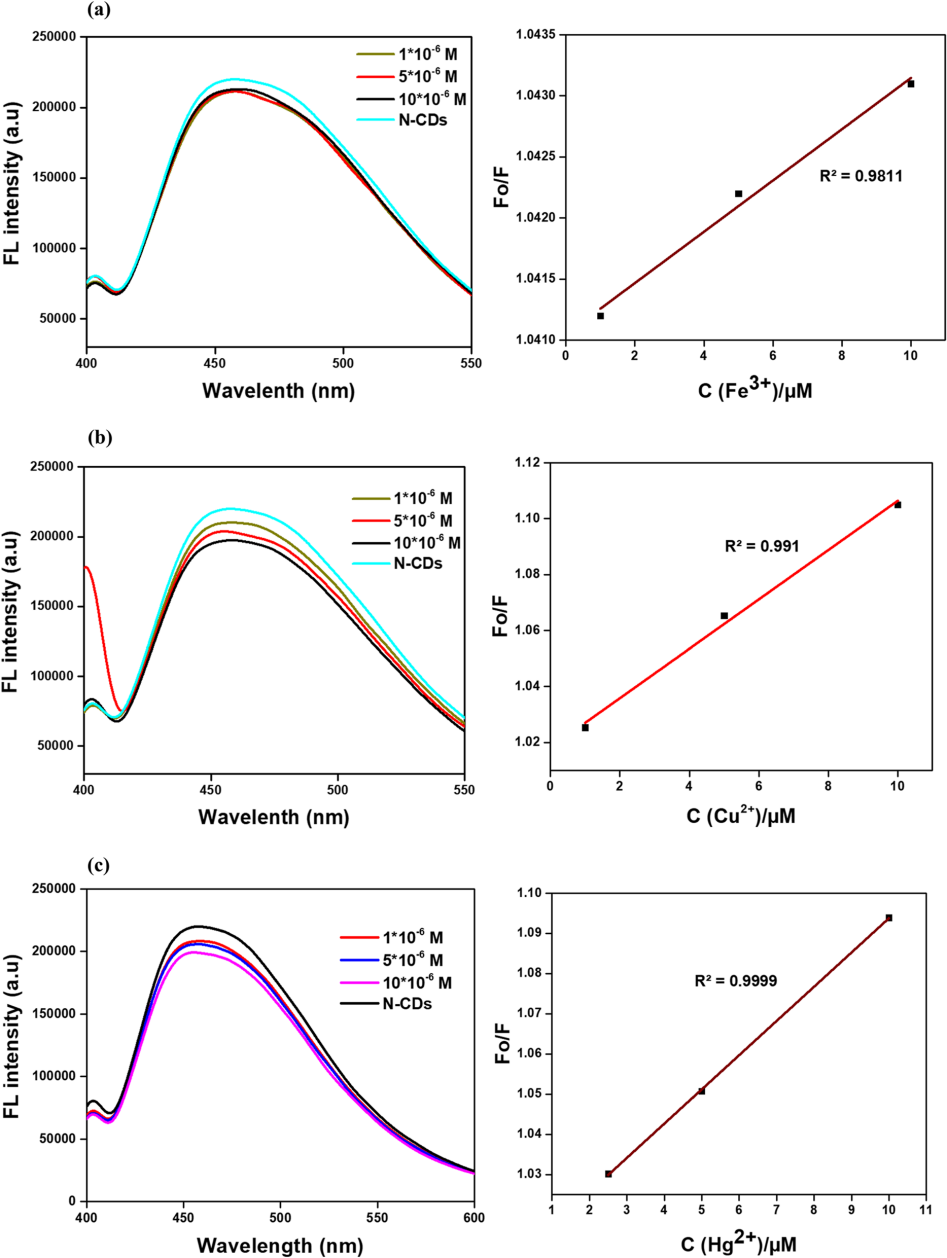
Determination of LODs of (**a**) Fe^3+^, (**b**) Cu^2+^ and (**c**) Hg^2+^ using the N-CDs

Until now, most research efforts have focused on enhancing the sensitivity and selectivity of CD-based fluorescence sensors for detecting heavy metals. In polluted water, metal ions can exist in different concentrations. High concentrations of metal ions can sometimes be spotted due to their color; however, they can be more dangerous at low concentrations by exceeding the relative environmental regulations but not showing any color. Therefore, investigating the limits of detection is a must. The determination of the LOD for metal ions was calculated using the formula

3 $$LOD=3 \sigma/\text{slope}$$

where sigma (σ) represents the standard deviation of the CDs blank sample without any quenchers, and the slope is derived from the calibration curve correlating fluorescence intensity with quencher concentration (Raveendran et al. 2019). The standard deviation σ was calculated to be 0.004 based on six blank samples of CDs without metal ions. The calibration curve of fluorescence quenching efficiency, expressed as F₀/F, demonstrated a strong linear correlation with Fe^3^⁺ concentrations ranging from 1 to 10 μM, achieving an excellent correlation coefficient (R^2^ = 0.99), as depicted in Fig. 8a.

The detection limit for Fe^3^⁺ was calculated to be 6 μM, which is close but, admittedly, still higher than the maximum permissible limit of Fe^3^⁺ in drinking water, which is 5.36 μM, as per WHO guidelines. As discussed in Sect. 3.4, Fe^3^⁺ ions cause fluorescence quenching in CDs through the IFE, where absorption overlaps with excitation wavelengths and reduces fluorescence intensity. This quenching at higher Fe^3^⁺ concentrations enables reliable detection of pollutants, but at lower concentrations, the mild quenching diminishes selectivity, making it harder to differentiate Fe^3^⁺ from other ions.

Consequently, the LOD for Cu^2^⁺ (Fig. 8b) was determined to be 1.36 µM, indicating the sensitivity of the CDs for detecting copper ions at low concentrations. For copper ions (Cu^2^⁺) in drinking water, Health Canada has established an aesthetic objective of ≤ 1.0 mg/L (1,000 µg/L) to address concerns such as staining and taste, as concentrations at this level are not typically considered a direct health risk. Similarly, the U.S. Environmental Protection Agency (EPA) has set a permissible safe limit of 1.3 ppm (20 µM) for Cu^2^⁺ in drinking water. Zhang et al. (2020a, b) synthesized CDs directly by combining Pu-erh tea with hot water and demonstrated an LOD for Cu^2^⁺ that was significantly lower than the permissible value recommended by the EPA for drinking water. The LOD for Hg^2^⁺, as depicted in Fig. 8c, was determined to be 1.41 µM, demonstrating the high sensitivity of the CDs towards low concentrations of Hg^2^⁺. For Hg^2^⁺, the maximum acceptable concentration is 0.001 mg/L (1 µg/L) due to its potentially harmful effects, such as neurological and kidney damage (Water 1996). The WHO sets a limit for mercury in drinking water as 0.006 mg/L (6 µg/L)​ (WHO 2022). Liu et al. (2019a, b) reported a LOD for Hg^2^⁺ that falls below this threshold, demonstrating the potential of their method for sensitive mercury detection in water. Since color changes usually happen at greater concentrations, copper ions would not significantly alter the color of the water at our detection limit for Cu^2^⁺ (1.36 µM, or around 86.7 µg/L). Similarly, as mercury usually does not cause noticeable color changes at low concentrations, mercury ions at the detection limit would not visually change the watercolor. This result confirms the effectiveness of CDs as fluorescent sensors for detecting Fe^3+^, Cu^2+^, and Hg^3+^ ions, offering significant potential for their application in environmental monitoring and analytical assays requiring precise quantification of trace metal ions.

### *Stern-Vomer* relationship for the interactions between N-CDs and metal ions

**Fig. 9:**
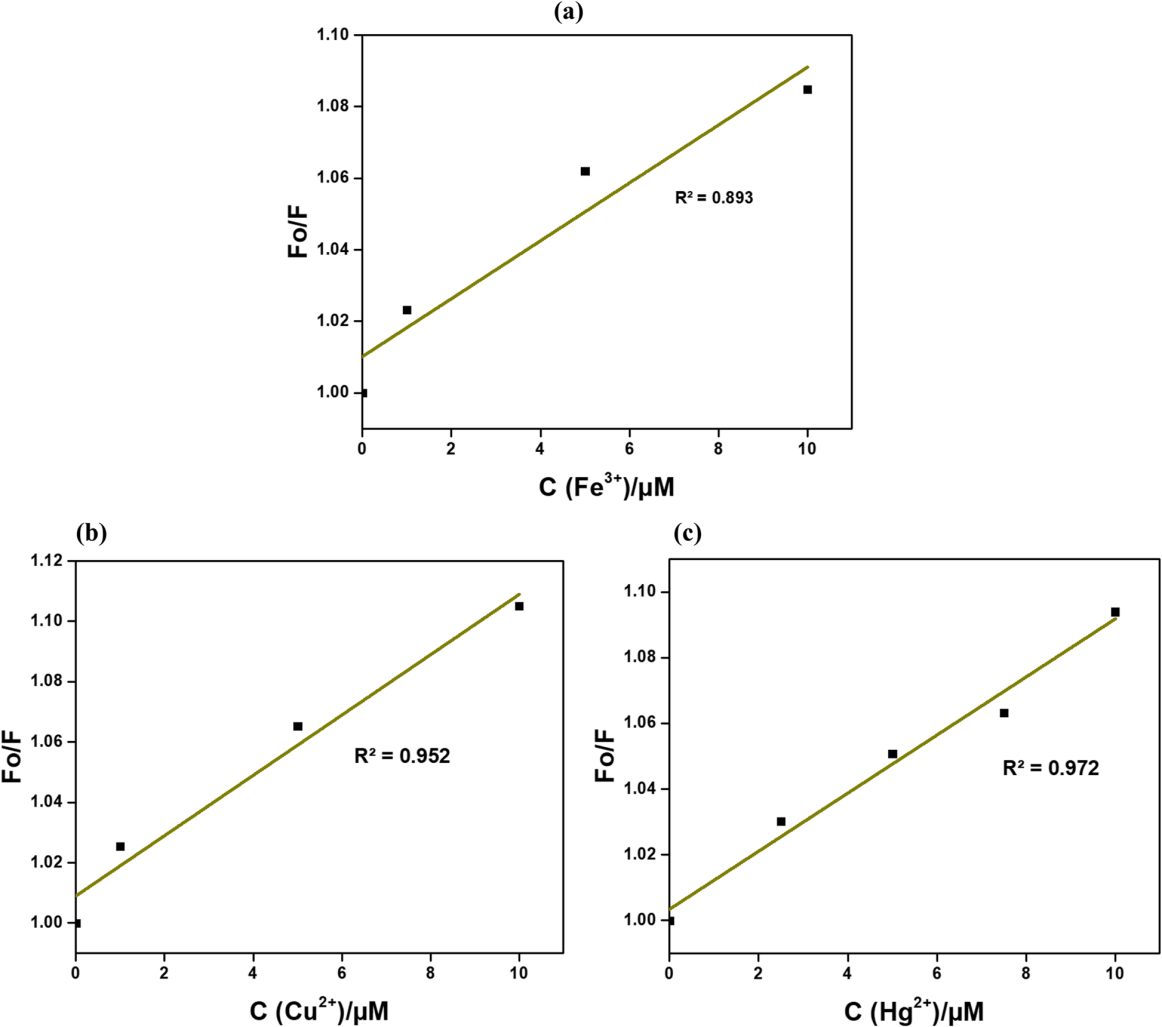
Data fit for *Stern-Vomer* equation for N-CDs (**a**) Fe^3+^, (**b**) Cu^2+^, and (**c**) Hg^2+^ ions

The *Stern–Volmer* equation is commonly used to analyze fluorescence spectroscopy data, quantifying the fluorescence quenching induced by analytes such as metal ions and providing insights into the kinetics of the quenching process. In this study, quenching constants for various metal ions were derived using the Stern–Volmer model, with the highest quenching observed upon the addition of Fe ^3+^, Cu^2^⁺, and Hg^2^⁺ ions to the CD solution, as shown in Fig. 9a-c. The linearity of the fluorescence intensity versus quencher concentration plot indicates a simple quenching mechanism, likely governed by static or dynamic interactions, whereas deviations from linearity suggest the involvement of more complex processes, such as static quenching. This approach enabled the detection of Fe ^3+^, Cu^2^⁺, and Hg^2^⁺ ions within the 0–10 µM concentration range, yielding *Stern–Volmer* quenching constants of 0.0233 µM, 0.0095 µM and 0.0111 µM, respectively. A higher Stern–Volmer constant indicates faster kinetics in N-CDs' association with metal ions. These findings show that CDs prepared in this study can be used for applications in environmental monitoring Fe ^3+^, Cu^2^⁺, and Hg^2^⁺ ions (Arvapalli et al. 2020).

### Stability analysis of N-CDs under different pH, NaCl concentrations, and irradiation

**Fig. 10:**
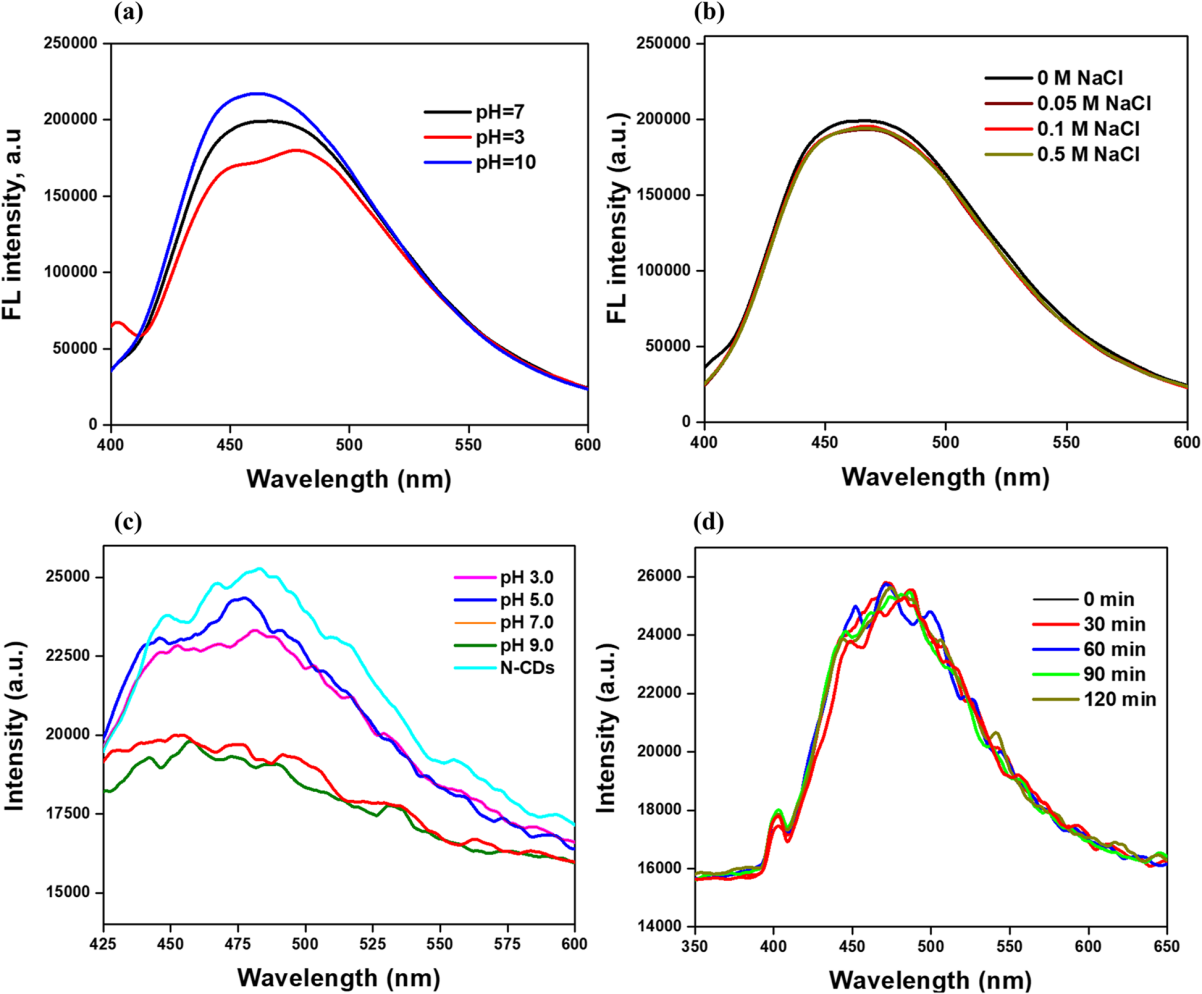
Stability analysis of N-CDs under different conditions: (**a**) pH variations, (**b**) salt concentrations, (**c**) Cu^2^⁺ solutions at different pH levels, and (**d**) FL spectra of N-CDs at various irradiation times under UV light (365 nm)

In real-life applications, the sample may be sourced from different water bodies with different properties (e.g., seawater); therefore, testing their stability under different pH and NaCl concentrations can help us understand their potential in dealing with such samples. The pH significantly influences the interaction process by altering the surface charge and dissociation of functional groups on N-CDs and affecting the chemical speciation of heavy metals (Zulfajri et al. 2019). To check the applicability of prepared N-CDs in seawater, the stability analysis under varying pH levels and sodium chloride (NaCl) concentrations was performed, as illustrated in Fig. 10a, b. The results indicated that adjusting the pH significantly influenced the emission intensity of the CDs, demonstrating the importance of pH adjustment in using their optical properties for metal ions sensing. Generally, the FL intensity increased when the pH increased from 3 to 10. In contrast, no substantial changes in emission intensity were observed across various salt concentrations, suggesting that the presence of NaCl does not markedly affect the stability or fluorescence characteristics of the CDs. In other words, the FL intensity decreases as the metal (Cu^2^⁺) solution pH increases from acidic to neutral and decreases from neutral to basic, as shown in Fig. 10c. At a high pH above 9.0, the copper salts precipitate. The fluorescence emission spectra of N-CDs (Fig. 10d) were recorded under continuous UV light (365 nm) at irradiation times of 0, 30, 60, 90, and 120 min to assess their photostability. Despite a slight reduction in emission intensity over time, the emission wavelength and spectral shape remained unchanged, indicating minimal photobleaching. This robust photostability is likely due to functional groups that enhance solubility and dispersibility, thereby reducing aggregation-induced quenching, as well as narrow size distribution and optimized surface chemistry that mitigate non-radiative decay pathways. These findings are consistent with observations by Atchudan et al. (2024), who reported stable photoluminescence in CDs derived from *Coccinia grandis* for up to 125 min. The impact of interfering cations such as Na⁺, K⁺, and Ca^2^⁺ was investigated at a low concentration of 0.01 M, and no significant effect on metal ion sensing was observed. However, at higher concentrations, these cations might have an adverse effect on metal ion sensing. These findings demonstrate the potential of the CDs produced in this study for monitoring metal pollutants in both freshwater and seawater.

## Conclusions

This study introduces a rapid and eco-friendly microwave-assisted synthesis of biomass-based N-CDs using CMC and glycine as novel carbon and nitrogen precursors for efficient heavy metal detection. The optimal fluorescence excitation wavelength was found to be at 400 nm, with a moderately high quantum yield of 31.6 ± 1.5%. The N-CDs exhibited sufficient selectivity and sensitivity toward Fe^3^⁺, Cu^2^⁺, and Hg^2^⁺ ions. Fluorescence quenching was influenced by pH, with the quenching mechanisms primarily driven by electrostatic interactions, π–π interactions, inner filter effects, and energy transfer. For Fe^3^⁺ ions, fluorescence quenching was attributed to the inner filter effect and electron transfer mechanisms, with significant quenching observed at higher concentrations, improving sensitivity, while mild quenching at lower concentrations resulted in reduced selectivity. The *Stern–Volmer* analysis revealed linear quenching for Fe^3^⁺, Cu^2^⁺, and Hg^2^⁺ ions within the 0–10 µM concentration range. The detection limits (LOD) for these ions were determined to be 6.0 µM for Fe^3^⁺, 1.41 µM for Cu^2^⁺, and 1.36 µM for Hg^2^⁺, respectively. The detection limit for Cu^2^⁺ was found well below the safe limits established by Health Canada (1 mg/L) and the U.S. EPA (1.3 ppm). However, due to the stringent 1 ppb limit for Hg^2^⁺ in drinking water, further optimization of the sensing process and enhancement of the CD performance are needed to improve sensitivity for low-concentration Hg^2^⁺ detection as per the standard. These optimizations can include surface functionalization, like tailoring surface oxygen moieties, such as carboxyl, hydroxyl, and amine groups, which improves coordination with metal ions, enhancing sensitivity and selectivity. These functional groups facilitate electron transfer and can form complexes with specific metal ions, leading to fluorescence quenching or enhancement. Multi-element co-doping is another possible strategy to increase fluorescence quantum yield and enhance sensing capabilities. For instance, nitrogen-fluorine (NF-CDs) and nitrogen-chlorine (NCl-CDs) have been explored to modify CDs electronic structure and intrinsic properties. This study highlights the potential of biomass-derived nitrogen-doped carbon dots (N-CDs) as effective sensors for heavy metal detection, offering a promising strategy for environmental protection and water quality monitoring. To ensure their practical applicability in real-world environmental monitoring, future research should prioritize enhancing the sensitivity and selectivity of these sensors. Additionally, validation with actual water samples, such as lake and tap water, is essential to confirm their effectiveness and reliability under diverse environmental conditions. Furthermore, a comprehensive structural analysis, including particle size distribution, crystallinity, and surface characteristics, is necessary to optimize the physicochemical properties of the synthesized N-CDs and improve their performance in sensing applications.

## Data Availability

Data will be made available at a reasonable request.
